# ZC3H15 promotes gastric cancer progression by targeting the FBXW7/c-Myc pathway

**DOI:** 10.1038/s41420-022-00815-x

**Published:** 2022-01-21

**Authors:** Jianbing Hou, Pan Huang, Chao Lan, Shengjun Geng, Minghao Xu, Yudong Liu, Hongbo Chang, Zhongze Wang, Hongyu Gu, Yi Wang, Guang Yang, Hongjuan Cui

**Affiliations:** 1 State Key Laboratory of Silkworm Genome Biology, Key Laboratory for Sericulture Biology and Genetic Breeding, Ministry of Agriculture and Rural Affairs, College of Biotechnology, Southwest University, Chongqing, 400716 China; 2 Cancer Center, Reproductive Medicine Center, Medical Research Institute, Southwest University, Chongqing, 400716 China; 3 The Affiliated Hospital of Southwest University (The Ninth People’s Hospital of Chongqing), Chongqing, China

**Keywords:** Gastric cancer, Tumour biomarkers

## Abstract

Zinc finger CCCH-type containing 15 (ZC3H15), a highly conserved eukaryotic protein, which was associated with several cellular processes and was ubiquitously expressed in various human tissues. Recent studies indicated that ZC3H15 was involved in tumorigenesis and may be a potential biomarker in hepatocellular carcinoma (HCC) and acute myeloid leukemia (AML). However, the biological function and molecular mechanism of ZC3H15 in gastric cancer (GC) have not been studied. In this study, we revealed that ZC3H15 was highly expressed in GC and high ZC3H15 expression was closely linked to poor survival of patients with GC. We found that ZC3H15 promoted cell proliferation, migration, and invasion by increasing c-Myc expression. Next, we found that ZC3H15 could modulate c-Myc protein stability by suppressing the transcription of FBXW7, which was mainly responsible for c-Myc degradation. Moreover, silencing of FBXW7 in ZC3H15-knockdown GC cells could partly abrogate the effects induced by ZC3H15 downregulation. Taken together, our data unearth the important roles of ZC3H15 in GC development and suggest that ZC3H15 may be a potential target for the treatment of GC.

## Introduction

Gastric cancer (GC) is a serious threaten for human life and health [1–3]. Various risk factors have been reported to play a critical role in the development of GC, such as familial inheritance, helicobacter pylori infection, and unhealthy dietary habits [4–6]. At present, primary treatments of GC include surgery, radiation, chemotherapy therapy, and targeted therapy. Early detection of GC is important for the overall prognosis of patients with GC [7]. However, early detection and diagnosis are not carried out effectively due to the poor understanding of the pathogenesis and molecular mechanism of GC [8]. Therefore, an in-depth investigation into the underlying mechanism and efficient molecular targets is still of great significance for the treatment of GC.

Ubiquitous expression of ZC3H15 was revealed by the Human Multiple Tissue Northern Blot in various normal human tissues [9]. ZC3H15, its gene locus is at human chromosome 2q32.1, is identified as an immediate early erythropoietin response protein due to it is likely ortholog to mouse immediate early response erythropoietin 4 [9–11]. ZC3H15 is a classical CCCH-type zinc finger protein, suggesting it may function as a transcription factor in cell signaling. In addition, ZC3H15 protein also contains a conserved DRG family regulatory protein (DFRP) domain, which is essential for the association of ZC3H15 with DRG [12]. Moreover, Gianni et al. have revealed the interaction of ZC3H15 with TRAF2, which was responsible for the activation of NF-κB signaling [13–17]. Indeed, ZC3H15 acts as an important role involved in cell proliferation, apoptosis, cell adhesion, and transcription, and dysregulation of ZC3H15 has been reported in hepatocellular carcinoma (HCC) and acute myeloid leukemia (AML) [18]. However, the biological role of ZC3H15 expression in GC is unclear.

The c-Myc oncoprotein, a multifunction factor that is frequently upregulated in varieties of human neoplasms, is related to many physiological progressions such as cell survival, chemoresistance, and tumorigenesis [19]. Owing to the critical role of c-Myc in modulating cellular pathways, its expression is tightly regulated. The regulatory mechanisms of c-Myc are majorly included transcriptional regulation, acetylation, phosphorylation, and proteasomal degradation [20, 21]. F-box and WD repeat domain containing seven (FBXW7), a known E3 ubiquitin ligase involved in ubiquitylation and proteasomal degradation of c-Myc, is an important tumor suppressor and is commonly dysregulated in human cancers [22, 23]. Expression of FBXW7 is negatively correlated with the tumor malignancy of human cancers [24, 25]. Therefore, a better understanding of the mechanisms of the regulation of FBXW7 expression may be effective in therapy against cancer.

In this study, we found that ZC3H15 was overexpressed in GC and high ZC3H15 expression was closely linked to poor survival of patients with GC. ZC3H15 modulated cell proliferation, migration, invasion, and tumorigenesis by increasing c-Myc expression. In addition, we found that ZC3H15 could increase the protein stability of c-Myc by inhibition of FBXW7 transcription. Taken together, these data indicated that ZC3H15 may be a potential therapeutic target for GC.

## Materials and methods

### Cell culture and transfection

The GES-1 cells, GC cell lines, and embryonic renal cell line 293FT were purchased from the American Type Culture Collection (ATCC, Manassas, VA, USA). The HGC-27 cells were cultured in MEM (Minimum Essential Medium) supplement with penicillin and streptomycin(P/S) and fetal bovine serum (FBS); The other GC cell lines and GES-1 cells were cultured in RPMI-1640 (Roswell Park Memorial Institute-1640) mediums supplement with P/S and FBS. 293FT cell line was cultured as previously described [26]. All cell lines were tested mycoplasma-negative. The MEM, RPMI-1640, and DMEM media, FBS, and antibiotics were obtained from Thermo Fisher Scientific, Inc. (Waltham, MA, USA).

Sequences of the Scramble, shZC3H15, and shFBXW7 were obtained from GenePharma Co., Ltd (Shanghai, China), and were listed as below:

Scramble: AGCACACTAGAACCATGTGAA

shZC3H15#1: CAGATCCCAAGTCTGTAGTAT

shZC3H15#2: CCTAGAATCAACAGGATGTTT

shFBXW7#1: CCAGTCGTTAACAAGTGGAAT

shFBXW7#2: CCAGAGAAATTGCTTGCTTTA

The shZC3H15#1 and shZC3H15#2 sequences were respectively used to target the coding region and untranslated region of the ZC3H15 gene. The FBXW7#1 and FBXW7#2 sequences were used to target the coding region of the FBXW7 gene. Vector encoding of human ZC3H15 and c-Myc were constructed by PCR-based amplification. Lentivirus was produced as previously described [26].

### Reagents

Dimethyl sulfoxide (DMSO) was obtained from Sigma-Aldrich (MO, USA). The c-Myc inhibitor 10058-F4 was purchased from Beyotime (Shanghai, China). The ZC3H15 (cat. no. NBP1-81312) antibody was purchased from Novus Biologicals (Shanghai, China); The Tubulin (cat. no. 11224) and HA (cat. No. 51064) antibodies were purchased from Proteintech (Wuhan, China); The FBXW7 (cat. no. ab109617) and c-Myc (cat. no. ab32072) antibodies were purchased from Abcam (Shanghai, China); Flag (cat. no. 14793) antibody was purchased from Cell Signaling Technology (Beverly, MA, USA).

### Immunohistochemistry staining

Tumor specimen was fixed by 4% paraformaldehyde for 24 h followed by dehydration. The tissues were embedded in paraffin and sectioned into 5μm thick sections, the sections were transferred on the glass slide and then 65 °C deparaffinized for 3–4 h followed by hydrated. The sections were performed by microwave heating for antigen retrieval, endogenous peroxidase incubation, and goat serum for blocking. After incubation for primary antibodies overnight at 4 °C and secondary antibodies for 2 h at room temperature, sections were covered with DAB (diaminobenzidine) for visualizing the staining. The staining was aborted by merging into the water. The sections were counterstained with hematoxylin for 6–8 min, differentiation with hydrochloric acid alcohol for 7 s, and bluing with ammonia for 1 min.

### Cell viability and proliferation assays

MTT assay was performed to examine the cell viability of indicated GC cell lines. Cells (1 × 10^3^ cells/ well) were cultured in the 96-well plates, before measurement, MTT was added into the medium for 2 h and DMSO was added to dissolve the formazan. The plate was then sent to measure the absorbance in 560 nm wavelength.

For BrdU staining, 1 × 10^5^ indicated cells were seeded into 24-well plates. After incubated with BrdU (Sigma) and fixed in 4% PFA, cells were treated with 1 mol/l HCl for increasing the permeability and 5% goat serum for blocking. Then, cells were incubated sequentially with anti-BrdU primary antibody overnight, and Alexa FluorR 488 secondary antibody for 2 h. DAPI (4',6-diamidino-2-phenylindole) was used for nuclear staining.

### Flow cytometry

For cell cycle detection, indicated cells were harvested and fixed in 75% ethanol at 4 °C for 24 h. Cell lysate was washed by PBS buffer and stained with RNaseA (Sigma) and Propidium Iodide (BD, San Jose, CA, USA) at 4 °C for 1 h, and then analyzed with the FACS C6 flow cytometry (BD, USA).

For cell apoptosis detection, indicated cells were harvested and washed by PBS two times. Centrifuge and replace the PBS buffer with binding buffer, followed by Annexin-V-FITC and PI staining with Annexin-V-FITC/PI Apoptosis Detection Kit (Yeasen, Shanghai).

### Western blot analysis and Co-IP

For western blotting assay, cells were lysed by ice-cold RIPA lysis buffer (Beyotime, Shanghai) for 1 h. The supernatant was harvested for further measuring its concentration by BSA reagents (Beyotime, Shanghai) in 560 nm wavelength. The amount of a particular supernatant was mixed up and boiled with loading buffer (Beyotime, Shanghai), and was followed by SDS-electrophoresis, primary antibody incubation, HPR-linked secondary antibody incubation, and exposure. The detail was performed as previously described [27]. The results of the western blot were analyzed by using ImageJ software. And the quantifications were calculated with methods of normalized vs. the tubulin loading control or the experimental group vs. the control group.

For Co-IP assay, cells were lysed in ice-cold IP lysis buffer (Sigma, USA) and then incubated on a rocker with indicated antibody as well as IgG at 4 °C overnight. After incubation with Protein A/G PLUS-Agarose (Beyotime, Shanghai). The loading buffer was added and boiled in the water bath kettle. The supernatant was resolved by sodium dodecyl sulfate-polyacrylamide gel electrophoresis analysis.

### Ubiquitination assay

For the ubiquitination assay, indicated plasmids were transfected into the 293FT cells. After 48 h, MG-132 (50 μg/ml, Selleck, Houston, TX, USA) was added into the medium for 8 h before harvesting. Cells were lysed and then performed following the same protocol used in Co-IP.

### Turnover assay

The cells were transfected with indicated plasmids, and then a final concentration (100 μg/ml) of CHX (cycloheximide) was added into the media. After harvesting at the indicated time points, the cells were lysed and analyzed by western blotting.

### Quantitative and RT-PCR

Total RNA was harvested from the indicated cells with Trizol reagent and then measured the concentration of total RNA. 2 μg RNA was reversely transcribed into cDNA by iScript cDNA Synthesis Kit (BioRad, #170-8891). The expression of mRNA was measured by using a Roche LightCycler Real-Time PCR System. Primers for RT-PCR assays were listed as Table 1.

**Table 1:** Primer pairs for real-time PCR and ChIP assays.

*Primer pairs for real-time PCR*	
ZC3H15-F	AACAAAATCCACGTCAGGTAGC
ZC3H15-R	TGCACATACTACAGACTTGGGA
c-Myc-F	GTCAAGAGGCGAACACACAAC
c-Myc-R	TTGGACGGACAGGATGTATGC
FBXW7-F	TAGAACCCCAGTTTCAACGAGA
FBXW7-R	GCCAACTCTTTAGGGAGCAAT
GAPDH-F	GGAGCGAGATCCCTCCAAAAT
GAPDH-R	GGCTGTTGTCATACTTCTCATGG
*Primer pairs for ChIP assays*	
FBXW7-1/-436-F	GTGCATAGATTGCCTTCCCAG
FBXW7-1/-436-R	CCATTCACAGTGCTCAATCAACTAT
FBXW7-372/-607-F	GACTGGCTGTTGGAAGAAGAAAATA
FBXW7-372/-607-R	ACGGCCTAAGATAAAGTCTGGAGAT
FBXW7-562/-835-F	GCCACTTTGAAGAGAGTCTTCATCT
FBXW7-562/-835-R	AAGCATAACAGTCACCCAACTGATT
FBXW7-804/-1020-F	TGTCTTTAATCAGTTGGGTGACTGT
FBXW7-804/-1020-R	ATGAGCACTATTTTCAAGTGTGTGC
FBXW7-1001/-1399-F	GAGAGCACACACTTGAAAATAGTGC
FBXW7-1001/-1399-R	AGTAATGTGAACACAACCAAAGCAG
FBXW7-1294/-1620-F	CTCCTCTTGGTTGACGAATACTCTC
FBXW7-1294/-1620-R	CTATGACGCGGGAGTTTAACAT

### Luciferase reporter assay

Cells were transfected with shZC3H15 or ZC3H15 together with the indicated reporter (FBXW7) or control plasmid. After 48 h, cells were harvested and lysed by Dual-Luciferase® Reporter Assay System (Promega, #E1910). The activity of firefly luciferase was detected by mixing 100 μL firefly luciferase reaction solution followed by adding 100 μL Renilla luciferase reaction solution and detecting for Renilla luciferase activity. The promoter fragments of FBXW7 were purchased from Wuhan GeneCreate Biological Engineering Co., Ltd.

### Chromatin immunoprecipitation

Indicated cells were harvested and lysed with ice-cold SDS lysis buffer followed by ultrasonic disruption (25 W, 18 s). The supernatant was isolated and incubated with agarose for removing the non-specificity combination. Indicated primary antibody was added spin overnight and agarose incubation for 4 h. The gene fragments were isolated by ChIP assays were performed using the EZ-ChIP^TM^ kit (Millipore, CA, USA) according to the manufacture’s protocol, and then detected by Roche LightCycler Real-Time PCR System. The primers used in ChIP assays are listed as Table 1.

### Soft agar assay

For the soft agar assay, 0.4 × 10^3^ cells were mixed with 0.6% agarose (Sigma-Aldrich, USA) in RPMI-1640 medium and then plated into 12-well plates containing a solidified bottom layer (0.3% agarose in medium). Colonies were imaged and calculated after 2 weeks of growth.

### Animal experimental procedures and tumor xenograft experiment

All animal studies were approved by the Institutional Animal Care and Use Committee of Southwest University. Four-week-old female nude mice were purchased from Beijing Animal Research Center and were housed in the SPF room. For the tumor xenograft experiment, mice were randomly divided into three groups. HGC-27 cells (1 × 10^6^) stably transfected with Scramble, shZC3H15-1, and shZC3H15-2 were subcutaneously injected into the mice on 18 November 2019. Isoflurane anesthesia system, which could help animals enter an anesthetized state faster and recover quickly, was used to reduce the pain of the mice. Isoflurane anesthesia is an inhalation general anesthesia, and the anesthesia-induction is stable, rapid, comfortable, fast recovery, good muscle relaxation, no sympathetic nervous system excitatory effect. In addition, isoflurane has a low metabolic rate in the liver, so it has little toxicity to the liver, and repeated use has no effect obvious side effects. Isoflurane was purchased from Reyward Life Technology Co., Ltd. (Shenzhen, China), and the concentration was MAC 1.6%. After subcutaneous injection, the mice were sterilized with 75% medical alcohol. The mice were observed and weighed every 3 days, and the feeding conditions were strictly standardized. The volume of tumors was calculated as follows: *V* = (length × width2)/2. Randomization and single blinding were used for measurement. Before the tumors were collected, the isoflurane anesthesia system was also used to reduce mice’s pain, and then the mice were killed by cervical dislocation and the tumors were harvested. The bodies of mice were frozen at −20 °C and then transferred to Laibite Biotech Inc. (Chongqing, China) for incineration.

### Transwell assay

For the transwell assay, cells in serum-free MEM or RPMI-1640 medium were seeded into the 24-well Boyden chambers (8μm pore size, Corning). MEM or RPMI-1640 Medium with 10% FBS was added to the lower chamber. Cells on the inner chamber were erased by cotton, cells on the outer chamber were fixed in 4% paraformaldehyde (PFA) and then stained with crystal violet. Then, Cells were imaged and calculated.

### Patient data analysis and patient tumor tissues

Bioinformatics analyses were performed using these specific programs: TCGA (https://cancergenome.nih.gov), UCSC Xena (https:// xena.ucsc.edu/public/), Oncomine (https://www.oncomine.org/) and Kaplan–Meier-plotter (http://kmplot.com/analysis/). Clinical samples were obtained from Chaoying Biotechnology Co., Ltd. (Henan, China). All the studies were approved by the Medical Ethics Committee of Tongxu County People’s Hospital of Henan Province. All of the patients were informed consent.

### Gene set enrichment analysis (GSEA)

To gain insight into ZC3H15 expression associated with the biological processes in GC, GSEA was performed using the Broad Institute GSEA version 4.0.3 software. The TCGA database was downloaded from UCSC Xena (https://xena.ucsc.edu/public/). The gene sets used for the enrichment analysis were downloaded from the Molecular Signatures Database (MsigDB, http://software.broadinstitute.org/gsea/index.jsp).

### Statistical analysis

All experiments were performed at least three independent experiments, and the quantitative data were expressed as mean ± SD. Two-tailed Student’s *t*-test was performed to calculate significance in an interval of 95% confidence, and a value of *P* < 0.05 was considered statistically significant, *P < 0.05, **P < 0.01, ***P < 0.001.

## Results

### ZC3H15 was upregulated in GC and high expression of ZC3H15 was correlated with poor patient prognosis

**Fig. 1:**
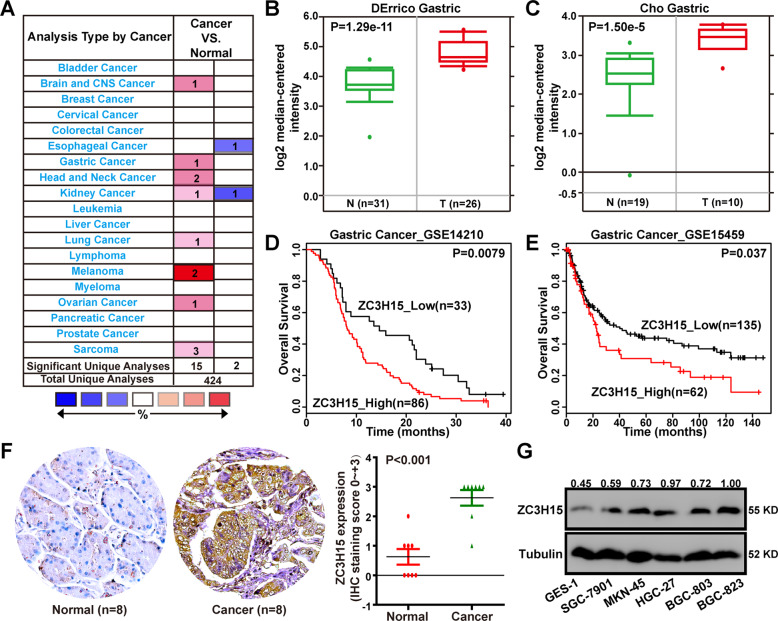
ZC3H15 was upregulated in GC and high expression of ZC3H15 was correlated with poor patient prognosis. **A** Up-regulation of ZC3H15 was found in 8 of 20 cancer types. **B**, **C** The level of ZC3H15 mRNA was significantly increased from normal stomach tissues to gastric cancer tissues in DErrico and Cho dataset and *P*-values were indicated. **D**, **E** Kaplan–Meier analysis of overall survival using data from the GSE14210 and GSE15459 database and *P*-values were indicated. **F** Immunohistochemical analyses of ZC3H15 expression in eight paired samples of gastric cancer and normal stomach tissue, *P* < 0.001. The data were expressed as mean ± SD. Student’s *t*-test was performed to analyze significance. **G** Western blot analyses were used to examine ZC3H15 expression in GES-1 cells and gastric cancer cell lines.

Overexpression of ZC3H15 was found in 8 of 20 cancer types through Oncomine data-mining analysis (Fig. 1A). In DErrico and Cho’s dataset from the Oncomine database, we found that the expression of ZC3H15 mRNA was significantly increased from normal stomach tissues to gastric cancer tissues (Fig. 1B, C). Then, we analyzed the expression data and survival information from the Gene Expression Omnibus (GEO) (GSE14210 and GSE15459), which was available from the Progression-free survival Kaplan–Meier database. We found that high ZC3H15 expression was significantly correlated with poor survival of GC patients (Fig. 1D, E). To confirm the expression of ZC3H15 in GC, we performed immunohistochemistry analysis (IHC) using primary tissue samples from GC patients. The results demonstrated that ZC3H15 expression was significantly higher in GC tissues (Fig. 1F). To confirm the role of ZC3H15 in GC, we analyzed the characteristics of GC patients related to ZC3H15 expression based on the TCGA database and the results indicated that ZC3H15 expression was significantly correlated with age, depth of invasion, and histologic grade of gastric cancer (Table 2). Then, we detected ZC3H15 expression at the protein level in human GC cell lines and normal gastric epithelial cells (GES-1). We found that ZC3H15 expression was commonly expressed in GC cell lines (Fig. 1G). Except for HGC-27, which was undifferentiated, all the other GC cell lines were low or moderately differentiated. Meanwhile, two cell lines SGC-7901 and BGC-823 were reported to be contaminated with Hela cells [28]. Hence, we used HGC-27 and MKN-45 cell lines to do the follow-up experiments. Taken together, these data indicated that ZC3H15 was upregulated in GC and high level of ZC3H15 was correlated with the poor prognosis of patients with GC.

**Table 2:** Correlation of ZC3H15 expression with clinicopathological variables in TGGA datasets.

Clinicopathological features		Cases	ZC3H15 expression				*F*	*P*
			Low	%	High	%		
Age	<60	134	79	59.0	55	41.0	8.009	0.005
	≥60	278	119	42.8	159	57.2		
Gender	Male	273	135	49.5	138	50.5	2.042	0.154
	Female	144	66	45.8	78	54.2		
*H. pylori* infection	Negative	164	56	34.1	108	65.9	0.499	0.481
	Positive	21	10	47.6	11	52.4		
Depth of invasion	T1	24	10	41.7	14	58.3	3.207	0.023
	T2	90	54	60.0	36	40.0		
	T3	189	86	45.5	103	54.5		
	T4	111	51	46.0	60	54.0		
Lymph node metastasis	N0	130	66	50.8	64	49.2	0.935	0.424
	N1	114	55	48.2	59	51.8		
	N2	86	38	44.2	48	55.8		
	N3	77	39	50.6	38	49.4		
Distant metastasis	M0	374	178	47.6	196	52.4	0.294	0.588
	M1	22	14	63.6	8	36.4		
Histologic Grade	G1	10	5	50.0	5	50.0	4.042	0.018
	G2	153	61	39.9	92	60.1		
	G3	245	131	53.5	114	46.5		
Grade	Stage I	58	31	53.4	27	46.6	1.738	0.159
	Stage II	138	71	51.4	67	48.6		
	Stage III	174	75	43.1	99	56.9		
	Stage IV	34	19	55.9	15	44.1		

### ZC3H15 knockdown inhibited the cell proliferation, migration, and invasion of GC cells

**Fig. 2:**
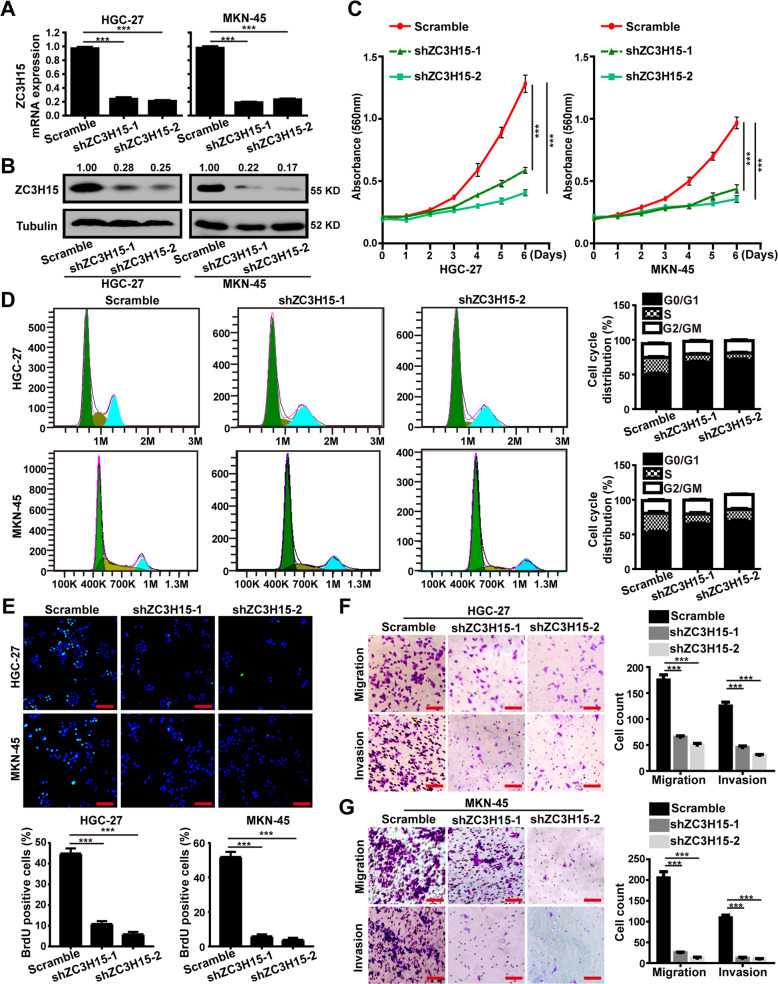
ZC3H15 knockdown inhibited the cell proliferation, migration, and invasion of GC cells. **A** The expression of ZC3H15 mRNA in ZC3H15-knockdown and control cells were detected by RT-PCR analysis. **B** The expression of ZC3H15 protein in ZC3H15-knockdown and control cells were detected by western blot analysis. **C** MTT assays were performed on stably transfected ZC3H15-knockdown and control cells. **D** Flow cytometry assays were performed to quantify the cell population in each phase of the cell cycle. **E** BrdU staining assays were performed to detect the amount of DNA synthesis in ZC3H15-knockdown HGC-27 and MKN-45 cells. **F**, **G** Transwell assays were used to detect the migration and invasion ability of GC cells. All data were expressed as mean ± SD. Student’s *t*-test was performed to analyzed significance, *P < 0.05, **P < 0.01, ***P < 0.001.

To investigate the biological function of ZC3H15 in GC cells, GSEA analysis using TCGA datasets was performed and indicated the positive association with cell cycle and metastasis in ZC3H15 high expression GC (Fig. S1A, B). Then, we established stably transfected ZC3H15-knockdown HGC-27 and MKN-45 cells for further investigation. RT-PCR and western blot analysis were conducted to confirm the efficiency of the knockdown system (Fig. 2A, B). Then, MTT assays demonstrated that silencing of ZC3H15 in HGC-27 and MKN-45 cells significantly inhibited cell proliferation (Fig. 2C). Next, flow cytometry analysis was performed and indicated that ZC3H15 downregulation induced the G1 arrest in GC cells (Fig. 2D). And BrdU assays showed that the DNA synthesis ability was significantly reduced in the ZC3H15-knockdown cells (Fig. 2E). We then explored whether ZC3H15 knockdown could induce cell death, we stained HGC-27 and MKN-45 cells with PI and annexin-V and then analyzed them with flow cytometry. The results demonstrated that silencing of ZC3H15 had no significant influence on the apoptosis in gastric cancer cells (Fig. S2). Then, transwell assays were performed and the results demonstrated that ZC3H15 knockdown dramatically suppressed cell migration and invasion in HGC-27 and MKN-45 cells (Fig. 2F, G). Therefore, these results indicated that ZC3H15 knockdown suppressed cell proliferation, migration, and invasion of GC cells in vitro.

### ZC3H15 recovery restored the cell proliferation, migration, and invasion of ZC3H15-knockdown GC cells

**Fig. 3:**
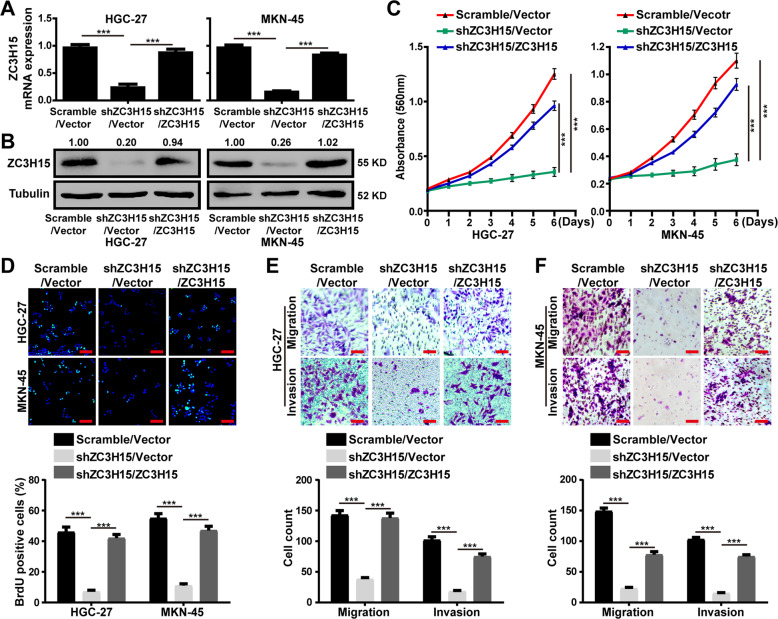
ZC3H15 recovery restored the cell proliferation, migration, and invasion of ZC3H15-knockdown GC cells. **A** RT-PCR assays were performed to detect the expression of ZC3H15 in indicated cells. **B** Western blot assays were performed to detect the expression of ZC3H15 in indicated cells. **C** MTT assays were performed to examine the effect of ZC3H15 overexpression on the proliferation of ZC3H15-knockdown cells. **D** BrdU assays were performed to examine the effect of ZC3H15 overexpression on the DNA synthesis ability of ZC3H15-knockdown cells. **E**, **F** Transwell assays were used to detect the effect of ZC3H15 overexpression on the migration and invasion of ZC3H15-knockdown cells. All data were expressed as mean ± SD. Student’s *t*-test was performed to analyzed significance, *P < 0.05, **P < 0.01, ***P < 0.001.

To further confirm the functional role of ZC3H15 in cell proliferation and metastasis of GC cells, we overexpressed ZC3H15 in ZC3H15-knockdown (shZC3H15#2) cells, in which ZC3H15 mRNA and protein expression levels returned to those in HGC-27 and MKN-45 cells (Fig. 3A, B). MTT and BrdU assays indicated that ZC3H15 overexpression could restore cell proliferation in the ZC3H15-knockdown HGC-27 and MKN-45 cells (Fig. 3C, D). Next, transwell assays demonstrated that ZC3H15 overexpression could rescue cell migration and invasion in ZC3H15-knockdown HGC-27 and MKN-45 cells (Fig. 3E, F). Taken together, these data demonstrated that ZC3H15 was essential for cell proliferation, migration, invasion of GC cells.

### ZC3H15 promoted GC progression by increasing c-Myc expression

**Fig. 4:**
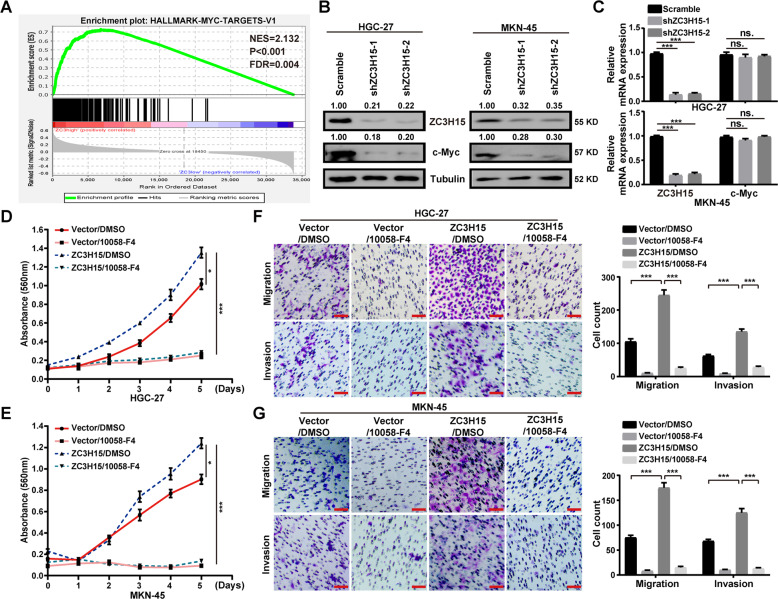
ZC3H15 promoted GC progression by increasing c-Myc expression. **A** GSEA enrichment plots of c-Myc target genes in high ZC3H15 expression versus low ZC3H15 expression TCGA GCs. Normalized enrichment score (NES), false discovery rate (FDR), and *P*-values were shown in the plot. **B**, **C** ZC3H15 modulated the protein and mRNA expression of c-Myc in HGC-27 and MKN-45 cells. **D**, **E** MTT assays were performed to examine the inhibitory effect of 10058-F4 (100 μM) on the cell proliferation of ZC3H15-overexpression HGC-27 and MKN-45 cells. **F**, **G** Transwell assays were performed to examine the inhibitory effect of 10058-F4 (100 μM) on the cell migration and invasion of ZC3H15-overexpression HGC-27 and MKN-45 cells. All data were expressed as mean ± SD. Student’s *t*-test was performed to analyzed significance, *P < 0.05, **P < 0.01, ***P < 0.001.

c-Myc signaling pathway is continuously activated in human cancers and was involved in malignant cancer progression, including migration, invasion, cell growth, and tumorigenesis. Through the GSEA analysis using TCGA datasets, we revealed that high ZC3H15 expression was positively associated with the c-Myc target genes in GC (Fig. 4A). To further confirm the effect of ZC3H15 knockdown on the expression of c-Myc in GC cells, we performed the western blot and quantitative PCR assays. We revealed that ZC3H15 positively regulated the protein expression of c-Myc in GC cells (Fig. 4B). However, the mRNA level of c-Myc has no significant change in ZC3H15-knockdown cells compared with control cells (Fig. 4C). These results suggested that ZC3H15 might post-transcriptionally regulate c-Myc. To confirm whether ZC3H15 regulated GC progression by targeting the c-Myc signaling pathway, we treated ZC3H15-overexpressing HGC-27 and MKN-45 cells with the c-Myc signaling inhibitor (10058-F4). The results demonstrated that the accelerated effects of ZC3H15 overexpression on cell proliferation, migration, and invasion were dramatically blocked by treated with 10058-F4 (Fig. 4D–G). In addition, we overexpressed c-Myc in ZC3H15-knockdown HGC-27 and MKN-45 cells. We found that the effects of ZC3H15 knockdown on cell proliferation, migration, and invasion were dramatically recovered after c-Myc overexpression treatment (Fig. S3A–D). Taken together, these data indicated that ZC3H15 promoted GC progression by increasing c-Myc expression.

### ZC3H15 stabilized c-Myc by mediating its ubiquitination degradation

**Fig. 5:**
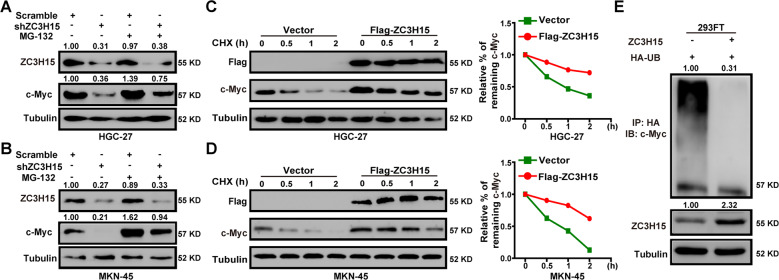
ZC3H15 stabilized c-Myc by mediating its ubiquitination degradation. **A**, **B** ZC3H15-knockdown HGC-27 and MKN-45 cells were treated with or without MG-132 for 8 h before harvesting. Western blot assays were performed to detect the protein expression of ZC3H15 and c-Myc. **C**, **D** The c-Myc turnover rate of ZC3H15-overexpression HGC-27 cells and MKN-45 cells was shown. Cells were treated with CHX (100 μg/ml) for the indicated times and then were harvested for western blot assays. **E** Transfected cells were treated with MG-132 for 8 h before harvesting. The ubiquitinated c-Myc proteins were pulled down with anti-HA antibody and immunoblotted with anti-c-Myc antibody. All data were expressed as mean ± SD. Student’s *t*-test was performed to analyzed significance, *P < 0.05, **P < 0.01, ***P < 0.001.

To further determine ZC3H15 regulated c-Myc protein at the post-transcription level, ZC3H15-knockdown HGC-27, and MKN-45 cells were treated with the proteasome inhibitor MG-132, and the results demonstrated that c-Myc downregulation could be partly rescued by MG-132 (Fig. 5A, B). We then examined the turnover rate of c-Myc and found that ZC3H15 overexpression in HGC-27 and MKN-45 cells significantly reduced the turnover rate of c-Myc (Fig. 5C, D). Moreover, the ubiquitination of c-Myc was detected by the ubiquitination assay, and the results indicated that ZC3H15 overexpression could reduce the ubiquitination of c-Myc (Fig. 5E and Fig. S4). Taken together, these data suggested that ZC3H15 regulated the stability of c-Myc through the reduction of c-Myc ubiquitination.

### ZC3H15 inhibited the transcription of FBXW7

**Fig. 6:**
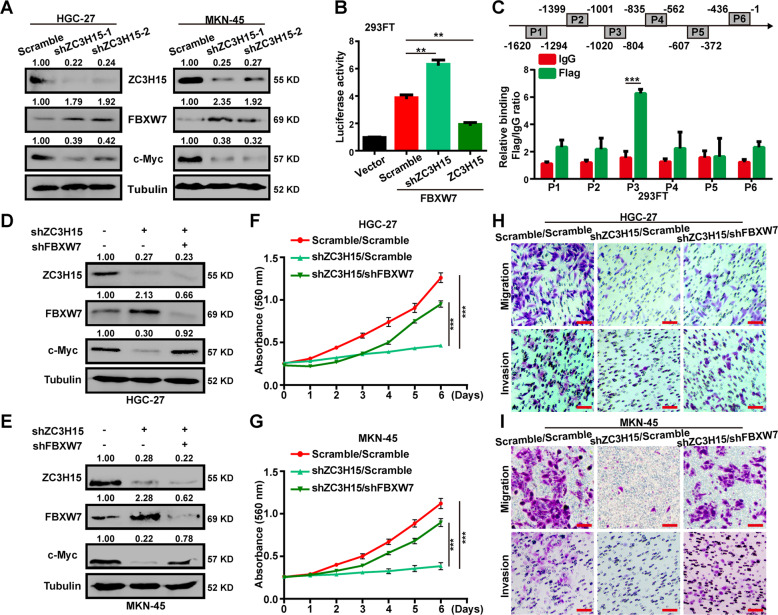
ZC3H15 inhibited the transcription of FBXW7 in GC cells. **A** Western blot assays were used to examine the protein expression of indicated cells. **B** Dual-luciferase reporter assays were used to detect the promoter activity of FBXW7. **C** ChIP assay was performed by using Flag antibody. IgG was used as the negative control. **D**, **E** Western blot assays were performed to detect FBXW7 and c-Myc protein expression of indicated cells. **F**, **G** MTT assays were performed to detect the effects of FBXW7 downregulation on the proliferation of ZC3H15-knockdown HGC-27 and MKN-45 cells. **H**, **I** Transwell assays were performed to detect the effects of FBXW7 downregulation on the cell migration and invasion of ZC3H15-knockdown HGC-27 and MKN-45 cells. All data were expressed as mean ± SD. Student’s *t*-test was performed to analyzed significance, *P < 0.05, **P < 0.01, ***P < 0.001.

FBXW7 is an important tumor suppressor and is responsible for the ubiquitylation and proteasomal degradation of c-Myc. ZC3H15 is a classical CCCH-type zinc finger protein, suggesting it may function as a transcription factor in cell signaling. Thus, we speculated that ZC3H15 might modulate the protein stability of c-Myc by targeting FBXW7. Then, we performed quantitative PCR and western blot analysis and found that the mRNA and protein expression levels of FBXW7 were negatively correlated with ZC3H15 in HGC-27 and MKN-45 cells (Fig. 6A and Fig. S5). Then, we performed the dual-luciferase reporter assay found that FBXW7 promoter activity was significantly enhanced in ZC3H15-knockdown cells and was reduced in ZC3H15-overexpressing cells, indicating that the promoter activity of FBXW7 was inhibited by ZC3H15 (Fig. 6B). To further determine whether ZC3H15 binds the promoter of FBXW7, we performed the ChIP assay and found that ZC3H15 bound the region P3 (-1020 to -804 bp) of the FBXW7 promoter (Fig. 6C). These data indicated that ZC3H15 could suppress FBXW7 transcription. To further confirm that ZC3H15 regulates the ubiquitination degradation of c-Myc by targeting FBXW7, we knockdown FBXW7 expression with the highly effective shFBXW7#2 in ZC3H15-knockdown HGC-27 and MKN-45 cells (Fig. S6). The results indicated that c-Myc expression was increased after FBXW7 knockdown in ZC3H15-knockdown cells (Fig. 6D, E). MTT assays were performed and indicated that the cell proliferation of ZC3H15-knockdown cells was clearly increased after FBXW7-knockdown treatment (Fig. 6F, G). In addition, silencing of FBXW7 also could promote cell migration and invasion of ZC3H15-knockdown cells (Fig. 6H, I). These data demonstrated that FBXW7 played as a critical downstream effector of ZC3H15 and that the ZC3H15-FBXW7 axis was responsible for c-Myc protein stability.

### ZC3H15 promoted the colony formation and tumor growth of GC cells

**Fig. 7:**
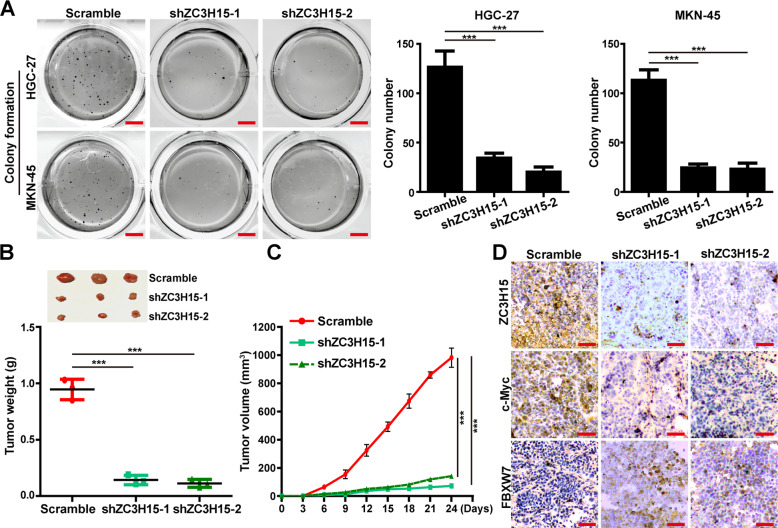
ZC3H15 promoted the colony formation and tumor growth of GC cells. **A** Soft agar assays were performed to detect the colony formation ability of GC cells. **B**, **C** Xenograft assays were performed in ZC3H15-knockdown HGC-27 cells. The weight and volumes of tumors were analyzed and *P*-values were indicated. **D** Immunohistochemical staining assays were performed to detect the expression of ZC3H15, c-Myc, and FBXW7 in ZC3H15-knockdown tumor tissues and control tissues. All data were expressed as mean ± SD. Student’s *t*-test was performed to analyzed significance, *P < 0.05, **P < 0.01, ***P < 0.001.

To explore the effects of ZC3H15 expression on colony formation of GC cells, soft agar assays were performed and demonstrated that ZC3H15 knockdown significantly reduced the colony formation ability of GC cells (Fig. 7A). To investigate the role of ZC3H15 in the tumor growth of GC cells, we performed the subcutaneous xenograft experiment and then found that ZC3H15 knockdown significantly retarded the tumor growth of GC cells (Fig. 7B, C). Immunohistochemical staining revealed that the expression ZC3H15 and c-Myc were dramatically reduced in the ZC3H15-knockdown tumors, whereas the expression of FBXW7 was elevated (Fig. 7D and Fig. S7). Taken together, these results demonstrated that ZC3H15 promoted tumorigenesis of GC cells by targeting the FBXW7/c-Myc signaling pathway.

## Discussion

GC exhibits high rates of proliferation and metastasis, which is a serious threat to human health. Gastrectomy is currently considered to be the mainstay of radical treatment. If the tumor is detected and treated in early diagnosis, the 5-year survival rate of GC can reach 90% [29]. However, the overall survival is extremely poor, with an average 5-year survival rate of <20% [30]. Therefore, a better understanding of the relationship between cancerogenesis, development, and prognosis will help to improve the diagnosis and treatment of GC.

In the present study, we found that ZC3H15 was upregulated in the patients with GC according to the immunohistochemistry and western blot analysis. Moreover, we observed that silencing of ZC3H15 inhibited cell proliferation, metastasis, and tumorigenesis of GC cells, and ZC3H15 overexpression could accelerate these progressions. These data suggest that ZC3H15 plays as an oncogene in GC cells. The biological mechanism of ZC3H15 in human cancers remains largely unclear. Bei et al. used a microarray to evaluate the functional role of ZC3H15, and they found that ZC3H15 was involved in several critical signaling pathways, such as the NF-κB pathway, EGF pathway, TGF-β pathway, and PDGF pathway [18]. Here, we demonstrated that ZC3H15 positively regulated the expression of c-Myc in GC cells. In addition, the protein expression of c-Myc could be partly rescued by using MG-132 in ZC3H15-knockdown GC cells. We then performed the ubiquitination assay and turnover assay and found that ZC3H15 positively regulated c-Myc protein expression through reducing c-Myc protein degradation. We then found that the mRNA expression of FBXW7, a well-known E3 ubiquitin ligase of c-Myc, was significantly elevated in ZC3H15-knockdown GC cells. In addition, we performed the dual-luciferase reporter assay and ChIP assay and found that ZC3H15 could inhibit the transcription of FBXW7 by binding to the promoter-proximal region P3 of FBXW7 promoter.

However, it is not clear that whether ZC3H15 binds directly to the genome or form a transcription complex and further binds to the genome, further studies are still needed. ZC3H15 contains two CCCH-type zinc finger domains and a DFRP domain. ZC3H15 is a classical CCCH-type zinc finger protein, suggesting it may function as a putative transcription factor role in cell signaling. If ZC3H15 binds directly to the genome, our study has indicated the binding range of the FBXW7, however, the exact binding sequence can be obtained by the combination of Electrophoretic Mobility Shift Assay and ChIP-sequencing assay, it will help us further understand and predict the genes it regulates, and also can help us find new targets. ZC3H15 also contains the DFRP domain, which was responsible for interacting with other proteins. Kosuke et al. reported that ZC3H15 interacted with DRG1 and then blocked the poly-ubiquitination and degradation of DRG1 [12]. Moreover, Gianni et al. reported that ZC3H15 regulated the NF-κB signaling pathways by interacting with TRAF2 [13]. Thus, it is necessary for further mass spectrometry analysis and protein structure analysis, it will help us to analyze and research the regulatory protein complex.

In this study, we illustrated the role of ZC3H15 that stabilized c-Myc protein level via inhibiting FBXW7 transcription to promote cancer progression. And we have obtained the combination sequence of FBXW7, and provided a new target for the drug development. Therefore, our research revealed the important roles of ZC3H15 in GC development and provided a brand-new insight for improving the prognosis for GC patients.

## Supplementary information

Figure-S1

Figure-S2

Figure-S3

Figure-S4

Figure-S5

Figure-S6

Figure-S7

Supplementary metarials

aj-checklist

cddiscovery-author-contribution-form

## Data Availability

All of the data and material in this paper are available when requested.
