# Supplementary figures and images for: ZC3H15 promotes gastric cancer progression by targeting the FBXW7/c-Myc pathway
Source: Cell Death Discov. 2022 Jan 21;8:32. doi: 10.1038/s41420-022-00815-x (PMC8782901; doi:10.1038/s41420-022-00815-x)

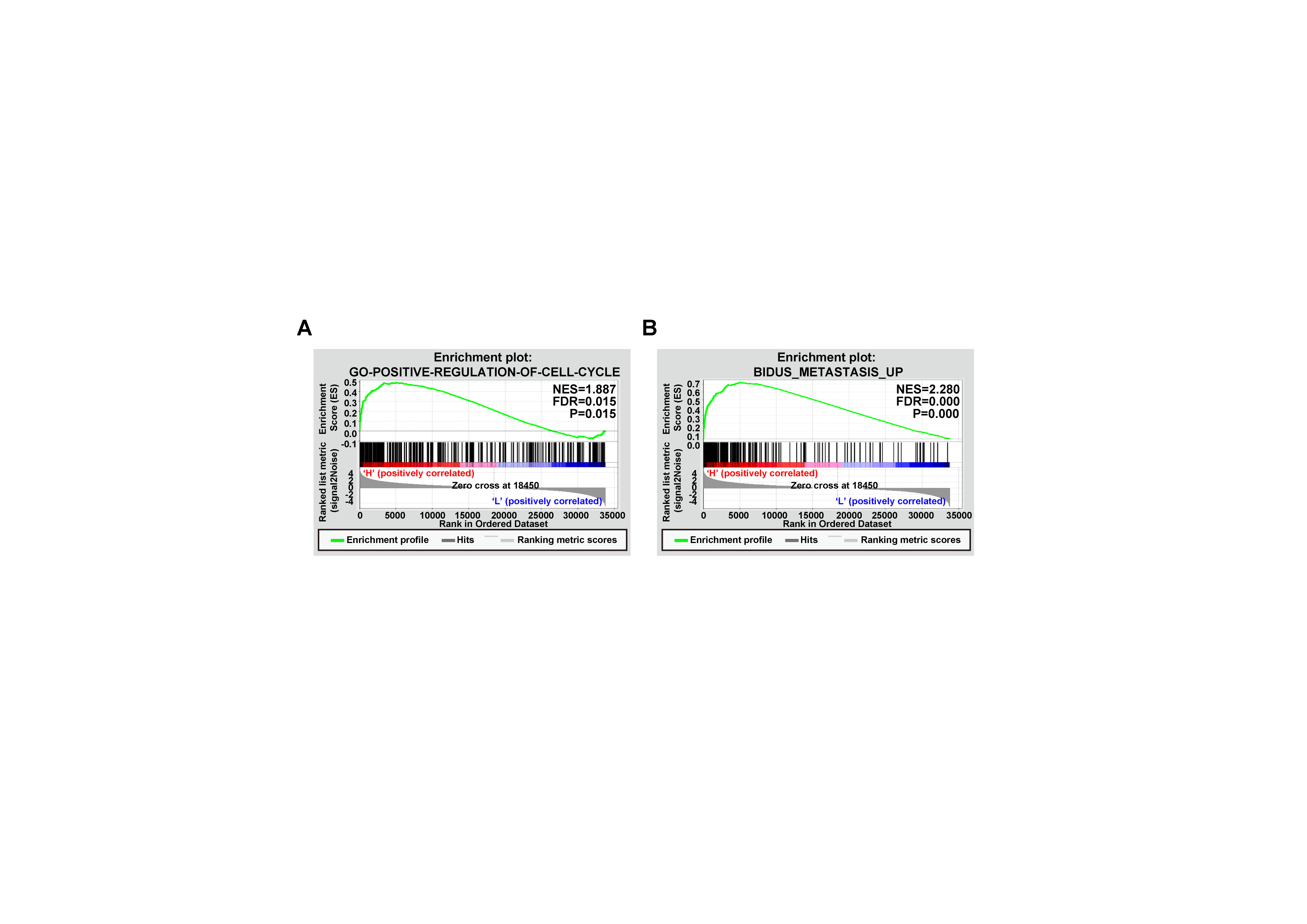

Supplement: Supplementary file 1 — Figure-S1 [file 41420_2022_815_MOESM1_ESM.tif]

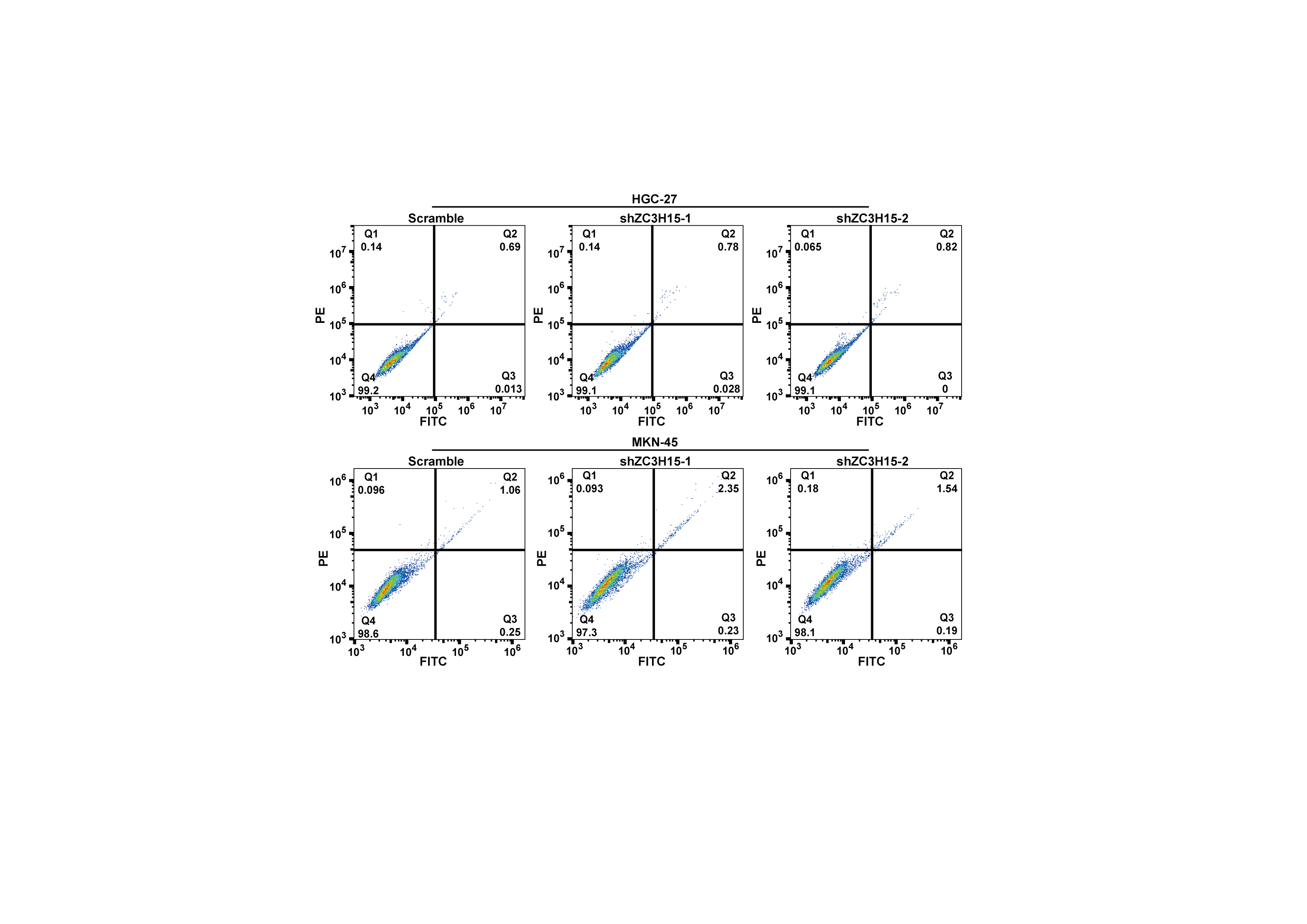

Supplement: Supplementary file 2 — Figure-S2 [file 41420_2022_815_MOESM2_ESM.tif]

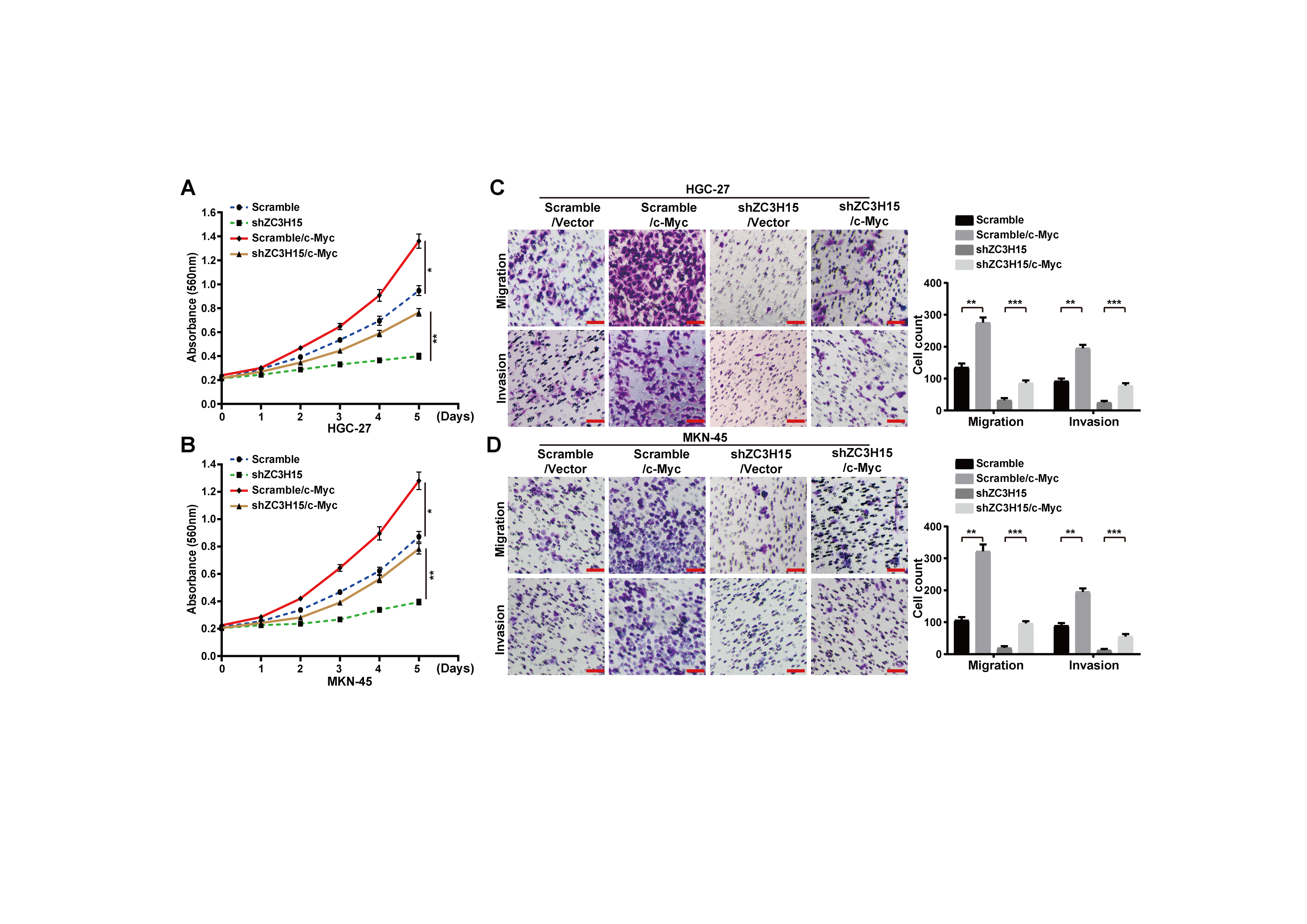

Supplement: Supplementary file 3 — Figure-S3 [file 41420_2022_815_MOESM3_ESM.tif]

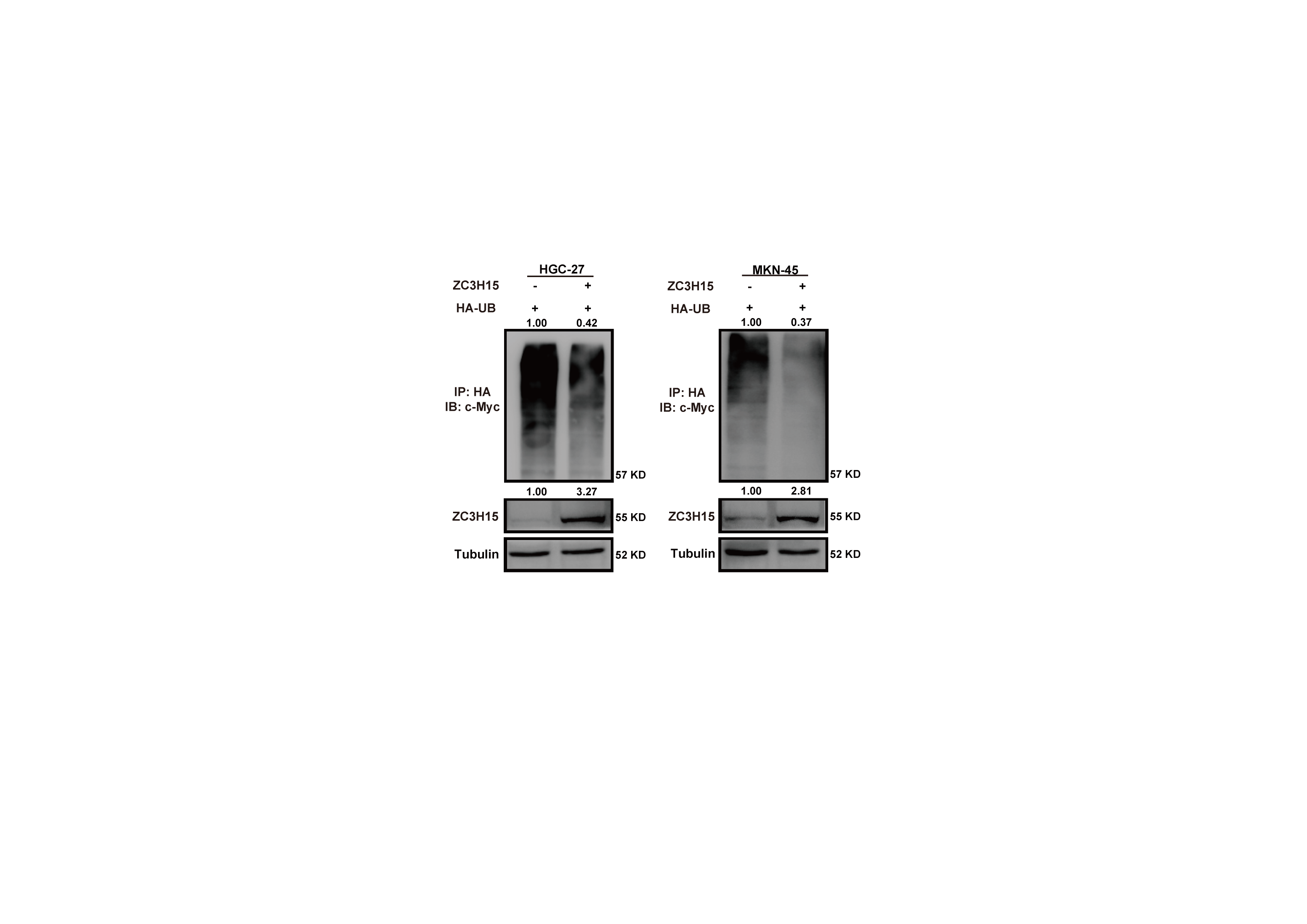

Supplement: Supplementary file 4 — Figure-S4 [file 41420_2022_815_MOESM4_ESM.tif]

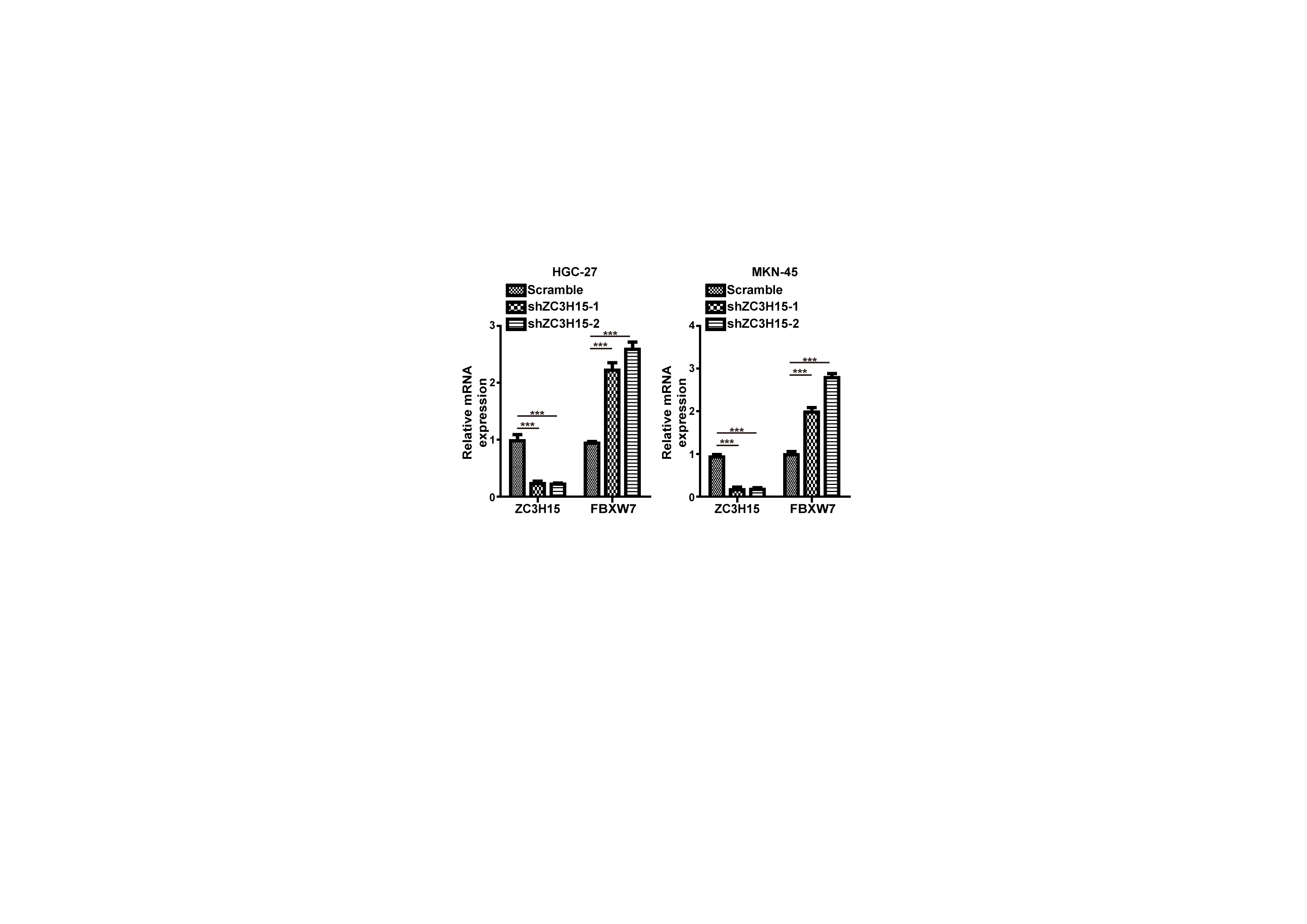

Supplement: Supplementary file 5 — Figure-S5 [file 41420_2022_815_MOESM5_ESM.tif]

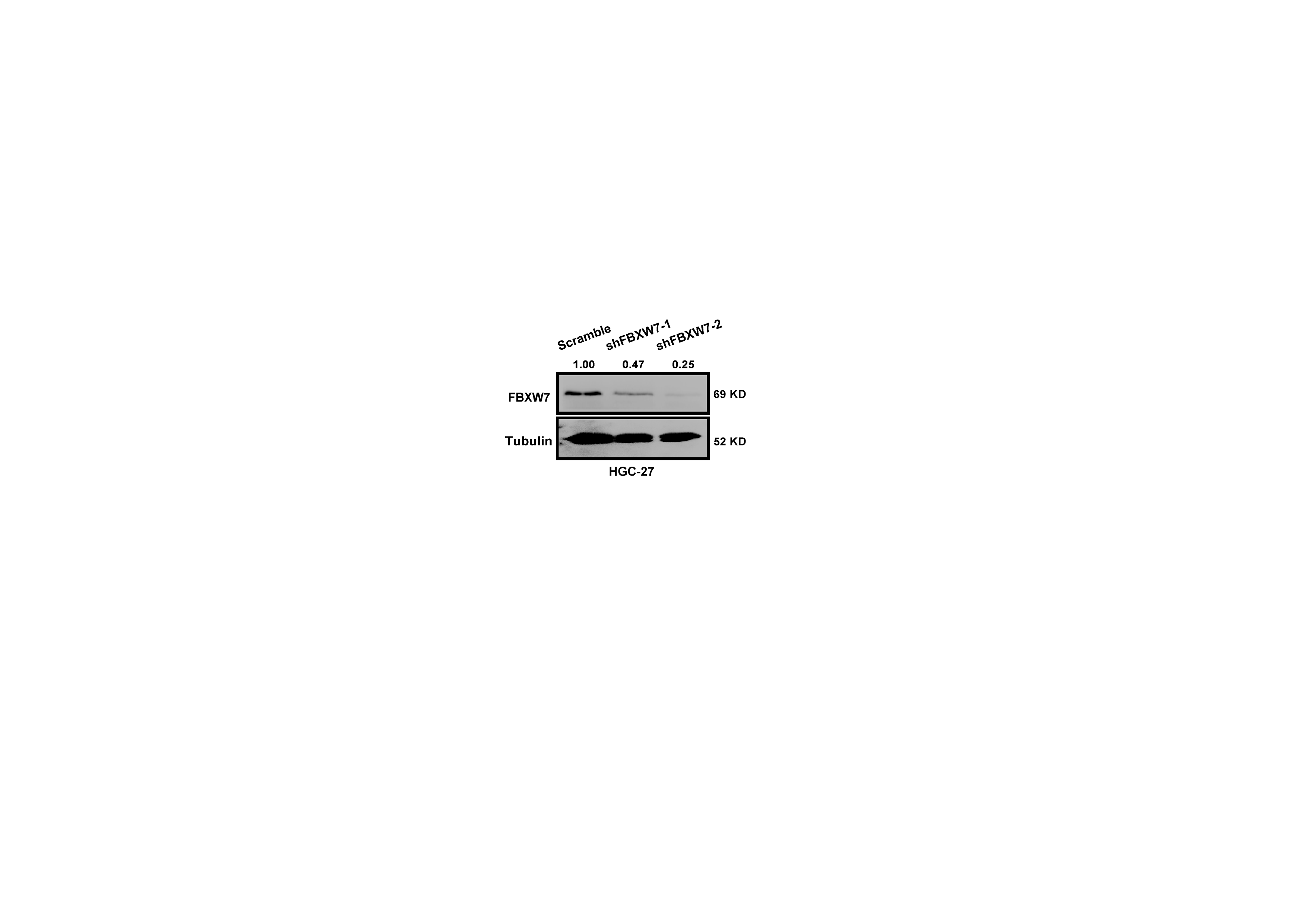

Supplement: Supplementary file 6 — Figure-S6 [file 41420_2022_815_MOESM6_ESM.tif]

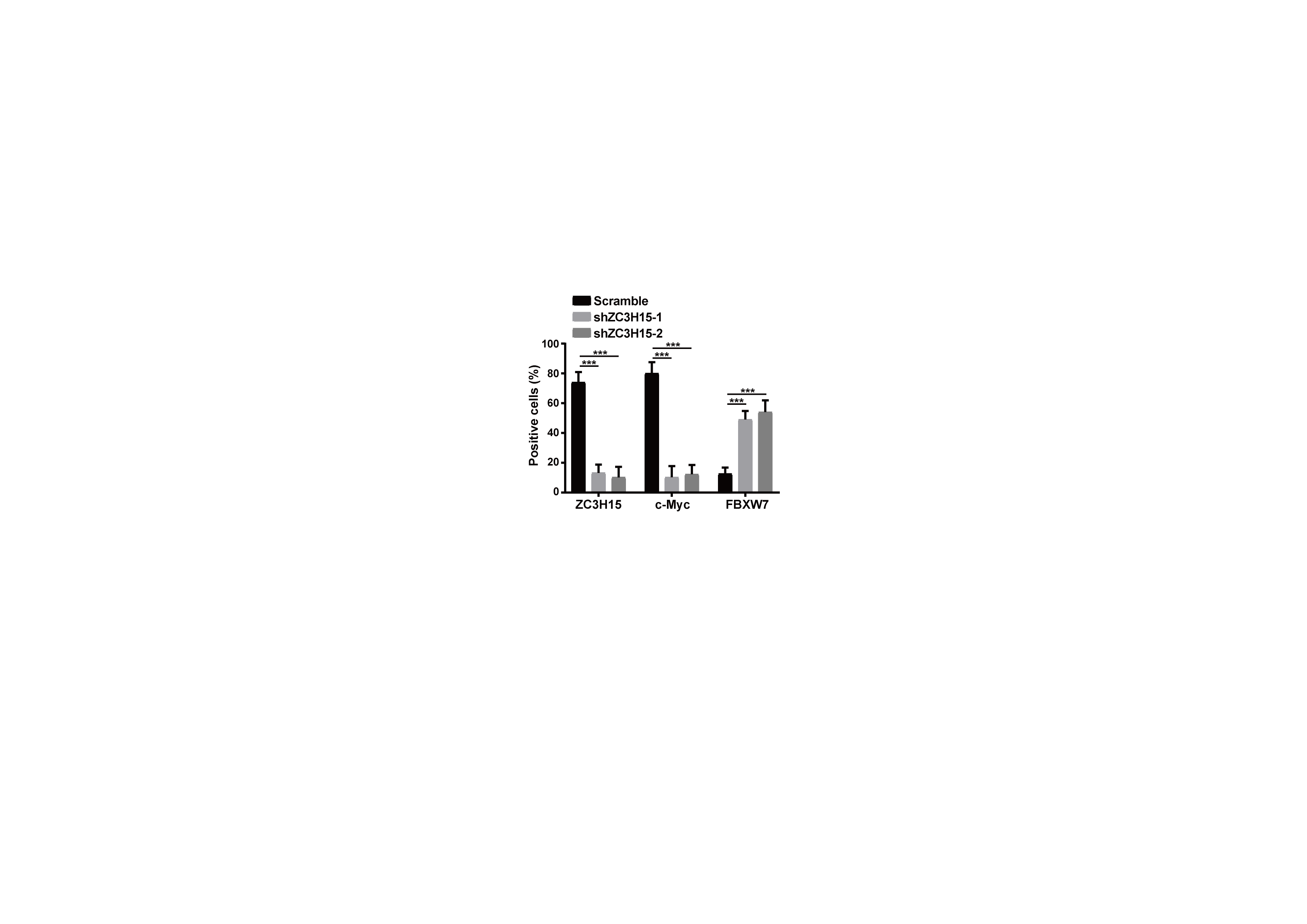

Supplement: Supplementary file 7 — Figure-S7 [file 41420_2022_815_MOESM7_ESM.tif]
