# Supplementary material for: ZC3H15 promotes gastric cancer progression by targeting the FBXW7/c-Myc pathway
Source: Cell Death Discov. 2022 Jan 21;8:32. doi: 10.1038/s41420-022-00815-x (PMC8782901; doi:10.1038/s41420-022-00815-x)
Supplement: Supplementary file 8 — Supplementary metarials [file 41420_2022_815_MOESM8_ESM.docx]

**Supplementary Fig. 1** (A, B) GSEA enrichment plots of cell cycle and metastasis signatures in high ZC3H15 expression versus low ZC3H15 expression TCGA GCs. Normalized enrichment score (NES), false discovery rate (FDR) and P-values are shown in the plot.

**Supplementary Fig. 2** Flow cytometric analysis of cell apoptosis in ZC3H15-downregulation and control cells.

**Supplementary Fig. 3** (A, B) MTT assays were performed to examine the effect of c-Myc overexpression on the cell proliferation of ZC3H15-knockdown HGC-27 and MKN-45 cells. (C, D) Transwell assays were used to detect the effects of c-Myc overexpression on cell migration and invasion of ZC3H15-knockdown HGC-27 and MKN-45 cells. All data were expressed as mean ± SD. Student’s t-test was performed to analyzed significance.

**Supplementary Fig. 4** The indicated plasmids were transfected into GC cells, and MG-132 was added to the cells before harvested. The ubiquitinated c-Myc proteins were pulled down with anti-HA antibody and immunoblotted with ant-c-Myc antibody.

**Supplementary Fig. 5** RT-PCR assays were performed to detect the mRNA levels of ZC3H15 and FBXW7 in ZC3H15-knockdown HGC-27 and MKN-45 cells. All data were expressed as mean ± SD. Student’s t-test was performed to analyzed significance.

**Supplementary Fig. 6** The expression of FBXW7 protein was detected by western blot analysis.

**Supplementary Fig. 7** The quantification of the positive cells in ZC3H15-knockdown tumor tissues and control tissues. All data were expressed as mean ± SD. Student’s t-test was performed to analyzed significance.
