# Supplementary material for: ZC3H15 promotes gastric cancer progression by targeting the FBXW7/c-Myc pathway
Source: Cell Death Discov. 2022 Jan 21;8:32. doi: 10.1038/s41420-022-00815-x (PMC8782901; doi:10.1038/s41420-022-00815-x)
Supplement: Supplementary file 10 — cddiscovery-author-contribution-form [file 41420_2022_815_MOESM10_ESM.pdf]

DECLARATION OF CONTRIBUTIONS TO ARTICLE

ADMC

Manuscript Number:	Journal Name:	
CDDISCOVERY-21-1988R1	*Cell Death Discovery*	(the 'Journal')

Proposed Title of the Contribution:

ZC3H15 promotes gastric cancer progression by targeting the FBXW7/c-Myc pathway (the 'Contribution)

Author(s):

Jianbing Hou, Pan Huang, Chao Lan, Shengjun Geng, Minghao Xu, Yudong Liu, Hongbo Chang, Zhongze Wang, Hongyu Gu, Yi Wang, Guang Yang*, Hongjuan Cui* (the 'Authors')

For all *CDDiscovery* articles, each person named as an author in the published version must be able to show he or she has contributed substantially to the article.

Authorship credit should be based on 1) substantial contributions to conception and design, acquisition of data, or analysis and interpretation of data; 2) drafting the article or revising it critically for important intellectual content; and 3) final approval of the version to be published. Authors should meet conditions 1, 2 and 3.

Any person who cannot be shown to have made a substantial contribution to the article cannot be listed as an author in the final version. The name of any person who is deemed to have made a minor contribution can, however, appear in the Acknowledgments section of the article.

Please complete the table below to indicate the contributions of all named authors to the manuscript.

Author Full Name:	Specification of Contribution to the Manuscript:
Jianbing Hou	Jianbing Hou designed experiments, obtained and analyzed data, and writed the manuscript.
Pan Huang	Pan Huang obtained and analyzed data, and revised the manuscript.
Chao Lan	Chao Lan obtained and analyzed data, and revised the manuscript.
Shengjun Geng	Shengjun Geng obtained and analyzed data, and revised the manuscript.
Minghao Xu	Minghao Xu obtained and analyzed data, and revised the manuscript.
Yudong Liu	Yudong Liu obtained and analyzed data, and revised the manuscript.
Hongbo Chang	Hongbo Chang obtained and analyzed data, and revised the manuscript.
Zhongze Wang	Zhongze Wang obtained and analyzed data, and revised the manuscript.
Hongyu Gu	Hongyu Gu obtained and analyzed data, and revised the manuscript.
Yi Wang	Yi Wang obtained and analyzed data, and revised the manuscript.
Guang Yang	Guang Yang designed experiments and revised the manuscript.
Hongjuan Cui	Hongjuan Cui designed experiments and revised the manuscript.

扫描全能王 创建

Please complete the table below to indicate the contributions of all named authors to the figures.

Figure 1:

In Figure 1, JH and PH analyzed the data and prepared panel A-E; CL, SG and MX generated immune-histochemistry data, labelled the image and prepared panel F; YL and HC generated the WB data and prepared panel G.

Figure 2:

In Figure 2, YL, HC, ZW and HG generated the WB and PCR data and prepared panel A and B;.JH, PH, CL,SG, MX and YW generated MTT, Flow cytometry, BrdU and transwell data and prepared panel C-G.

Figure 3:

In Figure 3, YL, HC, ZW and HG generated the WB and PCR data and prepared panel A and B;.JH, PH, CL,SG, MX and YW generated MTT, BrdU and transwell data and prepared panel C-F.

Figure 4:

In Figure 4, JH analyzed the data and prepared panel A; YL, HC, ZW and HG generated the WB and PCR data and prepared panel B and C;.JH, PH, CL,SG, MX and YW generated MTT and transwell data and prepared panel D-G.

Figure 5:

In Figure 5, JH, PH, YL, HC, ZW and HG generated the WB data and prepared panel A-E.

Figure 6:

In Figure 6, YL, HC, ZW and HG generated the WB data and prepared panel A, D and E; JH, PH and CL generated Luciferase reporter and ChIP data and prepared panel B and C; SG, MX and YW generated MTT and transwell data and prepared panel F-I.

In Figure 7, JH, PH and CL generated the colony formation data and prepared panel A; YL, HC, ZW and HG gerated the xenograft assay and prepared the panel B-D.

Signed for and on behalf of the Author(s):	Print Name:	Date:

扫描全能王 创建

Signed for and on behalf of the Authors:	Print Name:	Date:
Hong juan Cui	Hongjuan Cui	2021.11.16
Jianbing Hou	Jianbing Hou	2021.11.16
Pan Huang	Pan Huang	2021.11.16
Chao Lan	Chao Lan	2021.11.16
ShengJun Geng	Shengjun Geng	2021.11.16
Minghao Xu	Minghao Xu	2021.11.16
Yudong Liu	Yudong Liu	2021.11.16
Hongbo chang	Hongbo Chang	2021.11.16
Zhongze Wang	Zhongze Wang	2021.11.16
Hong yu Gu	Hongyu Gu	2021.11.16
Yi Wang	Yi Wang	2021.11.16
Guang Yang	Guang Yang	2021.11.16

扫描全能王 创建
